# Fuzzy logic controller for UAV with gains optimized via genetic algorithm

**DOI:** 10.1016/j.heliyon.2024.e26363

**Published:** 2024-02-15

**Authors:** Omar Rodríguez-Abreo, Juvenal Rodríguez-Reséndiz, A. García-Cerezo, José R. García-Martínez

**Affiliations:** a Space Robotics Laboratory, Department of Systems Engineering and Automation, Universidad de Málaga, C/Ortiz Ramos s/n, 29071 Málaga, Spain; b Facultad de Ingeniería, Universidad Autónoma de Querétaro, Querétaro 76010, Mexico; c Facultad de Ingeniería en Electrónica y Comunicaciones, Universidad Veracruzana, Poza Rica, Ver. 93390, Mexico

**Keywords:** Fuzzy logic, UAV, Metaheuristic algorithm, Genetic algorithm, Optimization

## Abstract

A gains optimizer of a fuzzy controller system for an Unmanned Aerial Vehicle (UAV) based on a metaheuristic algorithm is developed in the present investigation. The contribution of the work is the adjustment by the Genetic Algorithm (GA) to tune the gains at the input of a fuzzy controller. First, a typical fuzzy controller was modeled, designed, and implemented in a mathematical model obtained by Newton-Euler methodology. Subsequently, the control gains were optimized using a metaheuristic algorithm. The control objective is that the UAV consumes the least amount of energy. With this basis, the Genetic Algorithm finds the necessary gains to meet the design parameters. The tests were performed using the Matlab-Simulink environment. The results indicate an improvement, reducing the error in tracking trajectories from 30% in some tasks and following trajectories that could not be completed without a tuned controller in other tasks.

## 1 Introduction

Unmanned Aerial Vehicles (UAVs), also known as drones, have come to solve many problems, from the research of nature, health, security, industry, commerce, rescue, recreational, and service. Applications include inspection, supervision, monitoring, image collection, and cargo transportation [1]. Its ability to operate autonomously or be remotely controlled has opened up new possibilities in how people interact with our environment and carry out various tasks [2].

Various control techniques have been studied to control UAVs autonomously, such as nonlinear Proportional-Integral- Derivative (PID) used to stabilize the translational and rotational motion of a 6 degree of freedom (dof) UAV in [3], approaches based on the Lyapunov theory by the use of a backstepping based direct adaptive control for a quadrotor UAV [4], reference model-based integral sliding mode control method to control the velocity of a UAV [5] or a method used in [6] where the authors presented an altitude control algorithm for a DJI-F450 drone equipped with a laser range sensor. These works demonstrate the efficiency of the algorithms proposed to control UAVs and perform tracking trajectories with linear and non-linear controllers.

Fuzzy logic is an intelligent control system since it contains partially true and false states. Thus, defining operating rules that have shown many results in systems such as motors [7], robotic arms [8], or suspension systems [9]. The fuzzy logic applied to UAVs is observed in different areas. For example, in [10], the authors develop a fuzzy PID used to solve the problem of chattering caused by external disturbances, or in [11] where a fuzzy PI controller is implemented to adjust the parameters of a PI controller using the position and change of position data as input.

There are also fuzzy-logic works applied to the landing of UAVs using embedded systems, for example, the investigation shown in [12] where a Raspberry Pi for vision-based target detection method is utilized, and, besides, the integration of a PID and fuzzy logic controller are developed for safe landing. In addition, some research focuses on using fuzzy logic in UAVs applied to artificial vision [13] or UAVs designed using fuzzy logic to be fault tolerant [14]. Other authors focus their research on making adaptive fuzzy systems for UAVs to adaptively reduce the weight of disturbed acceleration and magnetic field measurements in the attitude estimation, as in [15].

In controller optimization, several works showed the benefits of tuning UAVs controllers. Although metaheuristic algorithms have been used in UAVs, as in the work presented in [16]. The study offers a solution that employs an Adaptive Neuro-Fuzzy Inference System (ANFIS) for identifying flight dynamics and utilizes genetic algorithms (GA) to optimize parameters for attitude control. In [17] an optimal direct, robust adaptive fuzzy controller for a quadrotor system using bat algorithm (BA) is presented. This approach aims to improve control by breaking down the system into four single-input and single-output subsystems. Each subsystem is controlled using a Mamdani-type fuzzy adaptive controller and a compensated control term based on tracking error. The adaptation parameters are optimized using the BA method. However, some authors explore the option of tuning the knowledge base of the fuzzy logic controller [18]. This option is an alternative in the optimization but requires more knowledge of the effect of the internal parameters in the controller. Although the work focuses on a controller based on error and its derivative, the proposed methodology can be extended to more complex controllers and made more precise or combined with knowledge-base tuning techniques in fuzzy logic controllers.

The most widely used metaheuristic algorithm is the Genetic Algorithm (GA). It has been widely used in multiple engineering problems with a high success rate [19]. For instance, [20] implemented a GA for online self-tuning with a focus on high-quality servo control and reduction of vibrations while positioning a linear motion system. [21] uses GA to optimize the values of the membership functions in the fuzzification of the input variables to a fuzzy controller in a servo system. The work presented in [22] used a GA to find the optimal flyable path for the UAVs in a 3D environment. Each generation is anticipated to be better than its previous generation in GA. On the other hand, a GA is employed in [23] to find sub-optimal coefficients of PID controller to optimize the performance of the closed-loop control system. [24] introduces a GA designed to optimize parameters for various autonomous flight control systems spanning a range of architectures and complexity levels; the authors proposed a comprehensive set of evaluation metrics tailored to assess the intricacies of autonomous flight trajectory tracking. These metrics were incorporated into a multi-objective fitness function, the guiding criterion for evolutionary selection. In the paper [25], the author discusses optimizing membership functions in a hierarchical Fuzzy Logic Controller for controlling a small autonomous parafoil used in reconnaissance and land survey missions. They manage the rule base size using a unique Combs method to prevent exponential rule expansion. Optimization is achieved through a steady-state genetic algorithm with a dynamic fitness function.

Previous works have shown the use of different algorithms to improve flight controllers. In some cases, fuzzy controller optimization processes are shown. However, the effect of gain tuning in fuzzy controllers using artificial intelligence has not been studied in any way. Unlike the publications presented, this work focuses on improving navigation results from metaheuristic tuning control gains. The results of this work show that the tuned controller can solve path-tracking problems that the untuned controller cannot solve.

The rest of the work is organized as follows: Section 2 presents the UAV model used. Section 3 exhibits the design of the fuzzy controller in its essential aspects and structure. Section 4 shows the Genetic Algorithm applied to the optimization of the controller. Section 5 shows the results of the tuned controller in tracking trajectories. Finally, the conclusions of the work are shown in section 6.

## 2 UAV modeling

In this section, the drone model used in subsequent tests is developed. A multirotor is an under-actuated system with six degrees of freedom. The drone model has been widely studied [26], which is why the model obtained for a multi-rotor UAV is summarized in Equation (1).

(1) $$\begin{array}{c} \overset{¨}{\phi }=\frac{I_{yy}-I_{zz}}{I_{xx}}(\overset{˙}{\theta }\overset{˙}{\psi })-\frac{lU_{2}}{I_{xx}}\\ \overset{¨}{\theta }=\frac{I_{zz}-I_{xx}}{I_{yy}}(\overset{˙}{\phi }\overset{˙}{\psi })+\frac{lU_{3}}{I_{yy}}\\ \overset{¨}{\psi }=\frac{I_{xx}-I_{yy}}{I_{zz}}(\overset{˙}{\phi }\overset{˙}{\theta })-\frac{U_{4}}{I_{zz}}\\ \overset{¨}{z}=(\cos\theta \cos\phi -g)\frac{U_{1}}{m_{T}}\\ \overset{¨}{x}=(\sin\psi \sin\phi +\cos\psi \sin\theta \cos\phi )\frac{U_{1}}{m_{T}}\\ \overset{¨}{y}=(\sin\psi \sin\theta \cos\phi -\cos\psi \sin\phi )\frac{U_{1}}{m_{T}} \end{array}\}$$

In this modeling way, two subsystems are clearly distinguished, the rotation one and the translation one, where $m_{T}$ is the mass of the drone. The variables that describe the linear position of the UAV for the global frame are *x*, *y*, and *z*. For the orientation of the UAV, the three principal angles are *ϕ*, *θ*, and *ψ*, and the $I_{xx}$, $I_{yy}$, and $I_{zz}$ are the inertia in the main axes. Finally, $U_{1}$, $U_{2}$, $U_{3}$, and $U_{4}$ are the control signals caused by motor speeds. The previous model takes the usual simplifications, such as the slight angle, and neglects the effects of gyroscopic torques and the ground effect. If the movement of the x−y plane is considered, two virtual control inputs, $U_{x}$ and $U_{y}$, are generated, which are used to calculate the pitch and bank angle necessary for the UAV to move in the x−y plane.

**Figure 1:**
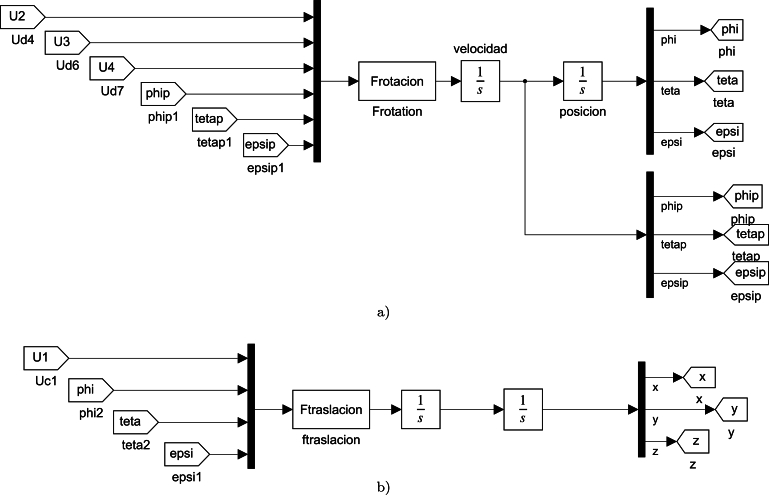
Simulink model used for simulated the UAV dynamic. (**a**) Model used for simulated the rotation subsystem; (**b**) Model used for simulated the translation subsystem.

The dynamic model is not necessary to use a fuzzy controller. However, the design process requires simulation tests that are not feasible in the physical system. For the simulation of the model, Simulink and an S-function were used to combine the calculation power of Matlab with the versatility of Simulink. An S-function was created for the UAV rotation (Fig. 1a)) subsystem and one for the translation subsystem (Fig. 1b)).

There are models that more accurately represent the model of a drone. However, the objective of the article is not modeling or control but is optimizing gains for a fuzzy control adaptable to dynamic models and more complex systems.

## 3 Fuzzy controller design

This section describes the design of a fuzzy controller that allows the system to follow a predetermined path. Therefore, the mathematical model defined by the set of Equations (1) was used to simulate the dynamics of the UAV. Six fuzzy controllers were designed in the Simulink-Matlab to control the complete system. Four controllers generated the control signals, which the method used directly (*z*, *ϕ*, *θ*, *ψ*). On the other hand, the remaining torques ($U_{x}$ and $U_{y}$) allowed the calculation of the reference pitch and roll angles, which, together with the control signals, will enable the system to follow the desired trajectory.

### 3.1 Fuzzy height control

The *z*-variable controller regulates the height of the Hexarrotor. The output of this controller returns the total power required by the rotors to correct the error. The difference in the desired altitude causes this error compared to the actual altitude of the Hexarrotor (Equation (2)).

(2) $$e_{z}=z_{r}-z$$

The derivative of the error was defined as (Equation (3)):

(3) $${\overset{˙}{e}}_{z}={\overset{˙}{z}}_{r}-\overset{˙}{z}$$

**Figure 2:**
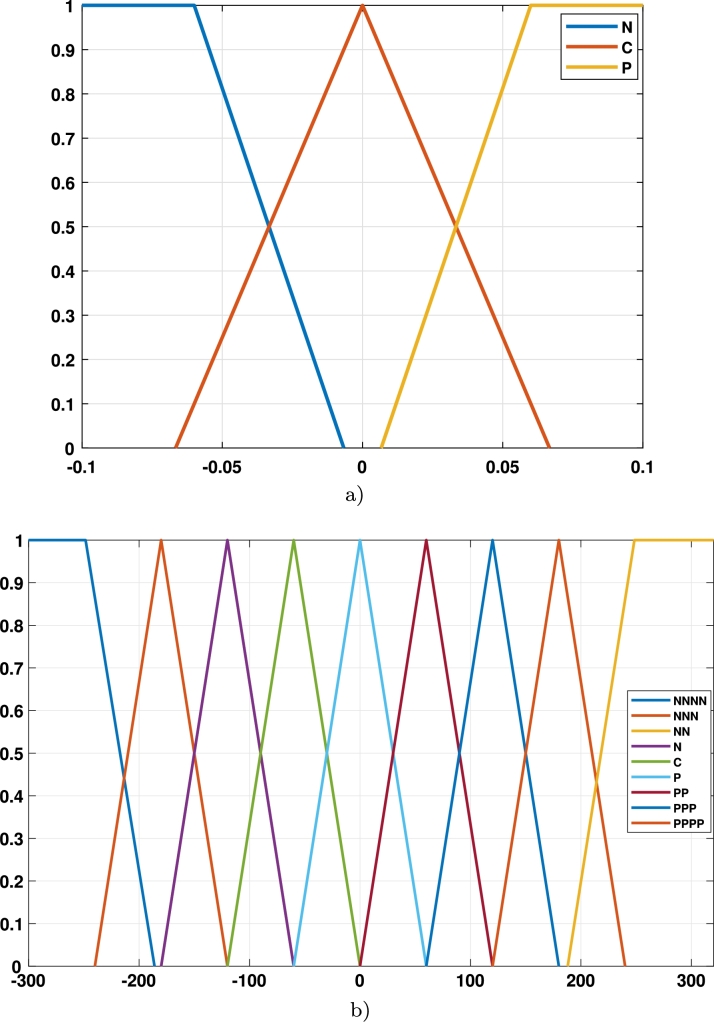
Membership functions in fuzzy controller (**a**) Input membership functions; (**b**) Output membership functions.

With these variables, a fuzzy controller can be designed for the z-axis of the UAV. For this controller, it was used three functions of membership for both the height error and its derivative, which represent a negative input (N), an input in a range close to zero (C), and a positive input (P), such as can be seen in Fig. 2a). First, for the fuzzification of the data, a degree of membership is assigned to each input variable concerning the membership functions previously defined in the fuzzy sets. Secondly, the output variable was defined with nine member functions from the most negative (NNNN) to the most positive (PPPP), as is displayed in Fig. 2b).

The chosen membership functions were triangular and trapezoidal type functions. The triangular type is appropriate for conditions with an optimal central value, which is lost when it is away from the center [27]. On the other hand, the trapezoidal function is employed in structures with a range of optimal values [27]. In this case, the triangular functions are suitable for the central and trapezoidal values for the extreme values. However, the design of the controller can be explored with other types of functions.

The values of the universes were selected using the magnitudes of the expected errors in the linear and angular positions and their derivatives. Therefore, different universes were taken according to the axis that was controlled. The X and Y controllers used the range of -0.5 to 0.5 m. The universe in the *ϕ*, *θ*, and *ψ* angle controllers are -90 to 90 degrees to cover the full range of angular motion. Finally, minor variations in the error are expected in the Z-axis. Thus, its universe varies from -0.1 to 0.1 m. The maximum expected rates of change were used to select the range in derivatives inputs.

After defining the inputs and outputs of the control system, it is necessary to dictate the rules that govern its behavior. The set of rules shown in Table 1 has been designed for this. In addition, the Mamdani-type fuzzy inference system and a defuzzification based on the centroid method were used.

**Table 1:** Height controller rules.

*Input* 1 (*e*_*z*_)	*Input* 2 (*e*_*z*_)	*Output* (*U*_1_)
N	N	NNNN
N	C	NNN
N	P	NN
C	N	N
C	C	C
C	P	PP
P	N	PPP
P	C	PPPP
P	P	PPPP

**Figure 3:**
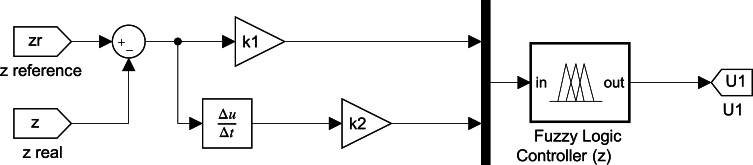
Structure of an axis fuzzy-controller.

Similarly, each of the six controllers was designed. The final structure of one of these controllers is shown in Fig. 3.

The parameters k1 and k2 are control gains and, by definition, must be greater than zero. Its value affects the performance of the controller. Therefore, each of the six controllers has two associated *k* gains. Non-linearities of the saturation type were added in each controller to keep the entries within the ranges of the defined universe.

## 4 Optimization of the gains via genetic algorithm

The gains of fuzzy controllers can be adjusted manually. However, a standard tuning technique does not exist, and the manual method has limited performance. Therefore, the metaheuristics algorithms are an option to find the optimum value for these gains in tracking trajectory tasks. The metaheuristic algorithm most widely used is the Genetic Algorithm. These algorithms are among a series of evolutionary algorithms recognized by the scientific community and used in various applications, such as particle swarm optimization (PSO), differential evolution (DE), and other bioinspired search methods. The description of how a Genetic Algorithm with Elitism works is displayed in Algorithm 1.

**Algorithm 1:**
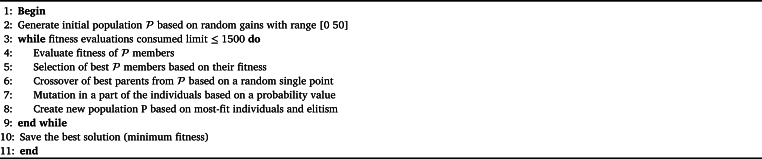
Genetic algorithm with elitism.

The Genetic Algorithm has various particular parameters depending on the type. In this work, the specific parameters of the Genetic Algorithm were Elitism, Mutation Probability, and Crossover Probability. Like all population-based algorithms, it requires the number of individuals, the stopping condition, the fitness function, and the search range for general parameters. Additionally, implementing the Genetic Algorithm is relatively simple, and the most significant computational cost is incurred in calculating the fitness. This characteristic is advantageous when working with high-cost computational systems such as the one in this work since an expensive algorithm would complicate the tuning of the gains. It should be noted that the fuzzy controller can be designed with different levels of complexity, increasing or decreasing the processing time of the tuning stage. However, tuning the same controller with metaheuristic algorithms will always have a higher computational cost when evaluating the fitness of each individual.

For this work, the general parameters used in the metaheuristic algorithm are shown in Table 2. The lower limit was selected because the gains are defined as positive constants. On the other hand, the upper limit was chosen as 50 since, with higher values, a saturation effect is observed. The stop limit was chosen at 50 since the RMSE value drops drastically in early iterations. However, in some cases, the RMSE decreased even after 30 or 40 iterations, so it was decided to stop at 50 to obtain the optimum values without extending the simulations.

**Table 2:** Parameter used in Genetic Algorithm.

Parameter	Value 2	Description
Population	30	Numbers of individuals in the search
Range of the search	[0 50]	Values between which best gains were searched
Stop condition	50	Number of iterations in which the search was done
Fitness function	$\sqrt{{\left(t_{s}-t_{sd}\right)}^{2}+{\left(M_{p}-M_{pd}\right)}^{2}}$	Function for evaluate the performance of proposed gains
Elitism	10%	Numbers of individuals in the search
Biological pressure	50%	Percentage of individuals that reproduce
Mutation probability	30%	Probability that individuals have a mutation

For the tuning, the dependent translation axis was grouped with their axis of rotation. x−ϕ, y−θ, and z−ψ formed three subsystems, and each subsystem has four associated gains to which they must be tuned together. The algorithm was executed three times, one for each subsystem, and the 12 gains that fulfill the characteristics of the desired response were found.

**Figure 4:**
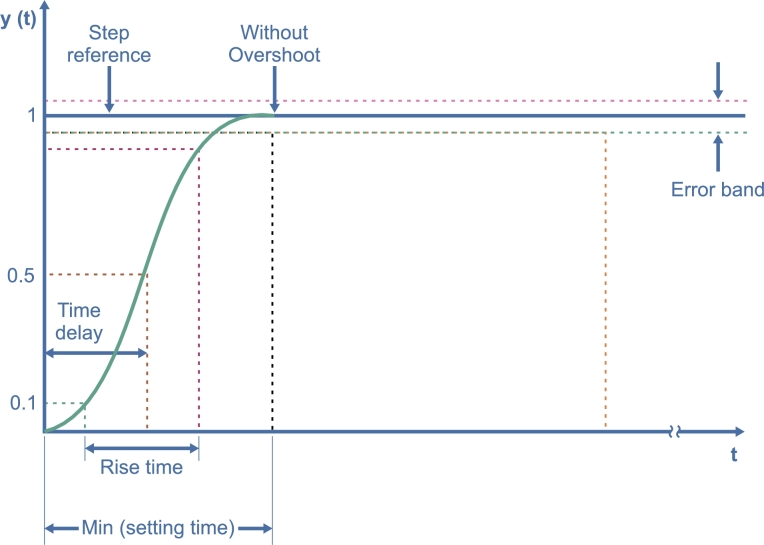
Ideal step response in UAV.

The desired response in an energy-limited system such as a UAV must reach the reference quickly without causing an overshoot. This guarantees that energy is not lost at low speeds or, on the contrary, that energy is lost in unnecessary braking (drone orientation changes). That is why the desired response is proposed with the following response characteristics to the step, as shown in Fig. 4.

In the tuning process, the principal characteristics for optimization are 0% overshoot but with a faster settling time and minimal steady-state error. The proposed reference signal complies with these characteristics. The transfer function shown in Equation (4) described the reference.

(4) $$Reference=step\left(\frac{5.22}{s+5.22}\right)$$

The Genetic Algorithm found the gains that minimize the Euclidean distance between the overshoot error and the establishing time error. The obtained gains and the features of the dynamic response are displayed in Table 3.

**Table 3:** Gains and dynamic response parameters obtained by Genetic Algorithm.

Axis	Gains obtained	ts	Overshoot
*z* − *ψ*	*k*_1_ = 3.401, *k*_2_ = 44.803, *k*_3_ = 0.421, *k*_4_ = 3.874	0.801 s	0.15%
*x* − *ϕ*	*k*_5_ = 0.068, *k*_6_ = 0.729, *k*_7_ = 0.9178, *k*_8_ = 0.490	0.843 s	0.4%
*y* − *θ*	*k*_9_ = 0.15, *k*_10_ = 0.81, *k*_11_ = 0.91, *k*_12_ = 0.46	0.729 s	0.31%

**Figure 5:**
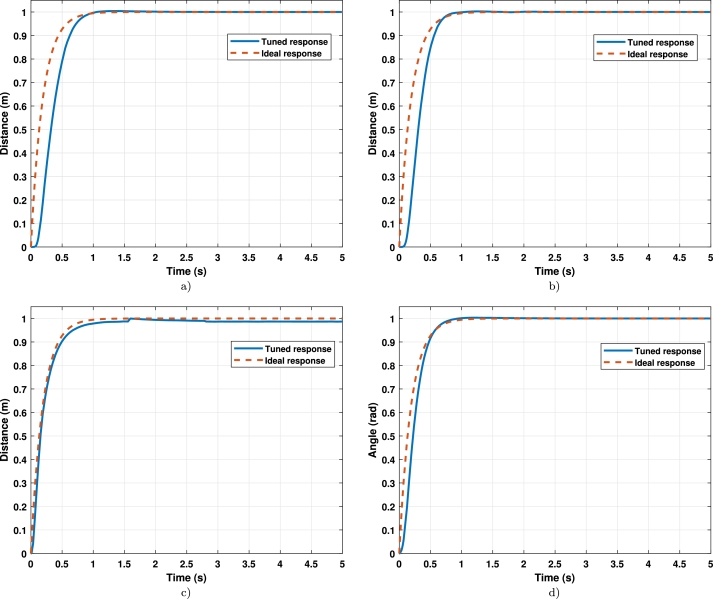
Step response of the tunned controller (**a**) X-axis response; (**b**) Y-axis response; (**b**) Z-axis response; (**b**) Ψ-axis response.

On the other hand, the graphic results are shown in Figs. 5a), 5b), 5c), and 5d) for *x*, *y*, *z*, and *ψ* respectively.

The results exhibit that the design parameters are met. Therefore, they are considered relevant results and must be tested in more complicated tracking trajectory tasks.

## 5 Results

This section shows the results of the adjustment of the gains. For this, the UAV is subjected to the monitoring of two trajectories. The first of these trajectories is described by set of Equations (5):

(5) $$\begin{array}{c} x=step\left(\frac{1}{{\left(s+1\right)}^{6}}\right)\text{ (in second 40) }\\ y=step\left(\frac{1}{{\left(s+1\right)}^{6}}\right)\text{ (in second 20) }\\ z=step\left(\frac{1}{{\left(s+1\right)}^{6}}\right)\text{ (in second 2 to 70) }\\ \psi =step\left(\frac{1}{{\left(s+1\right)}^{6}}\right)\text{ (in second 2) } \end{array}$$

The set of Equations (6) describes the second trajectory:

(6) $$\begin{array}{c} x=0.5cos(0.8t)\\ y=0.5sin(0.8t)\\ z=\frac{t}{5}\\ \psi =\frac{\pi }{3} \end{array}\}$$

**Figure 6:**
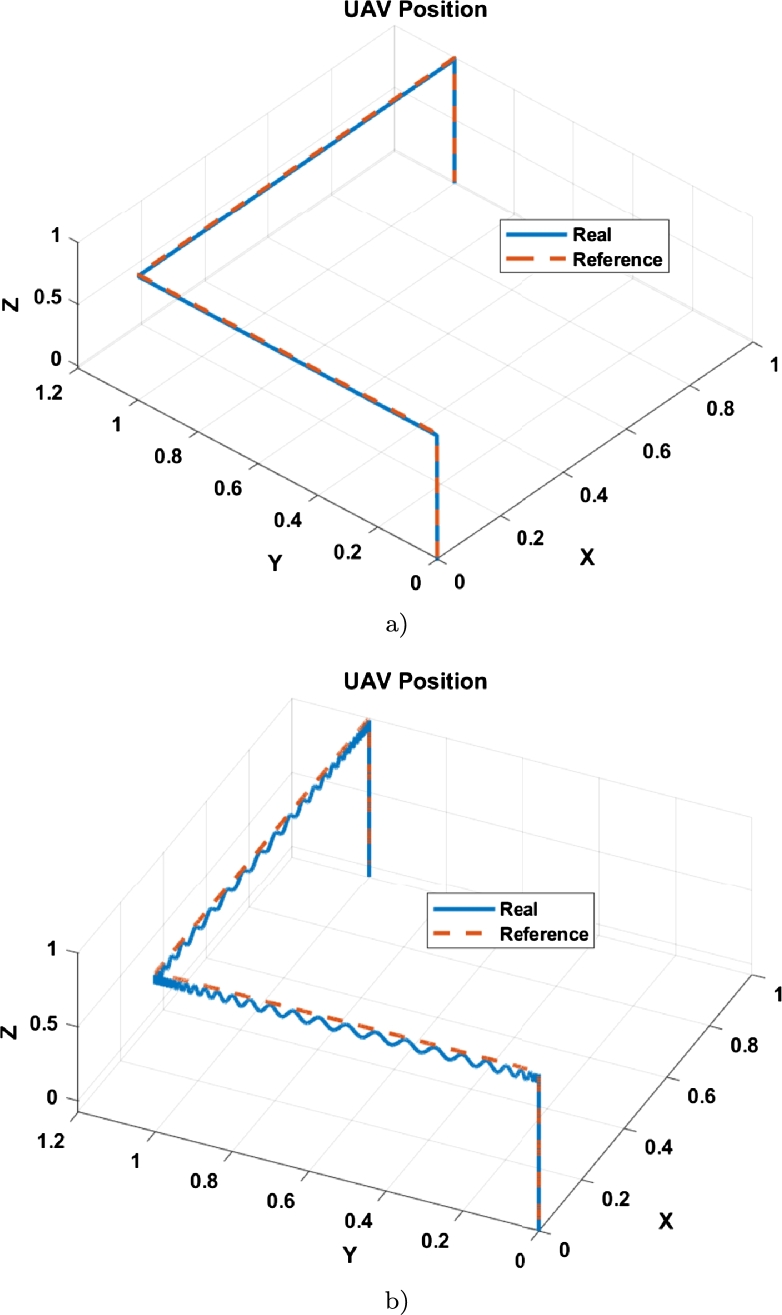
Position of the UAV in tracking the trajectory 1 (**a**) Tracking with tuned controller; (**b**) Tracking with untuned controller.

Both simulations were run for 90 seconds. The result of the tracking of the first trajectory is observed in Fig. 6, where the tracking is compared with the adjusted gains (Fig. 6a)) without adjusting them (Fig. 6b)).

**Figure 7:**
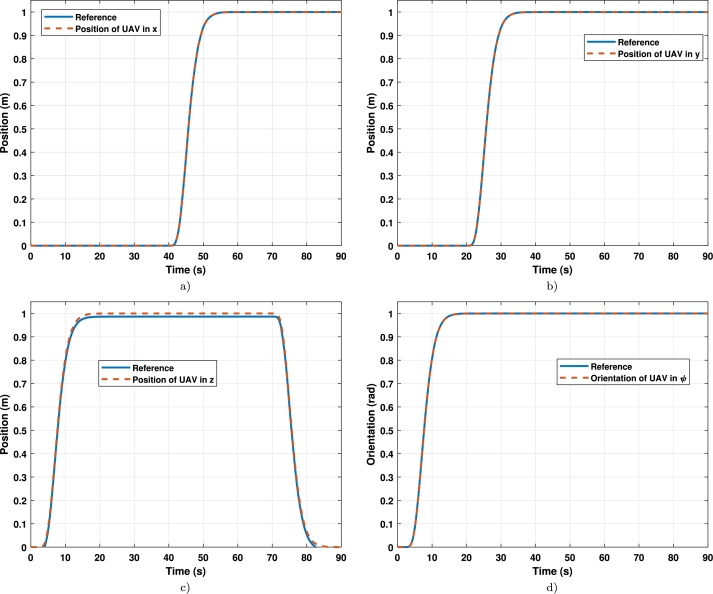
Position per axis of the UAV in tracking the trajectory 1 (**a**) x-axis vs reference; (**b**) y-axis vs reference; (**c**) z-axis vs reference; (**d**) *ψ*-axis vs reference.

The results per axis in tracking path 1 with tuned gains are shown in Fig. 7, where Fig. 7a) is the axis x, Fig. 7b) is the axis y, Fig. 7c) is the axis z, and Fig. 7d) is the yaw angle.

**Figure 8:**
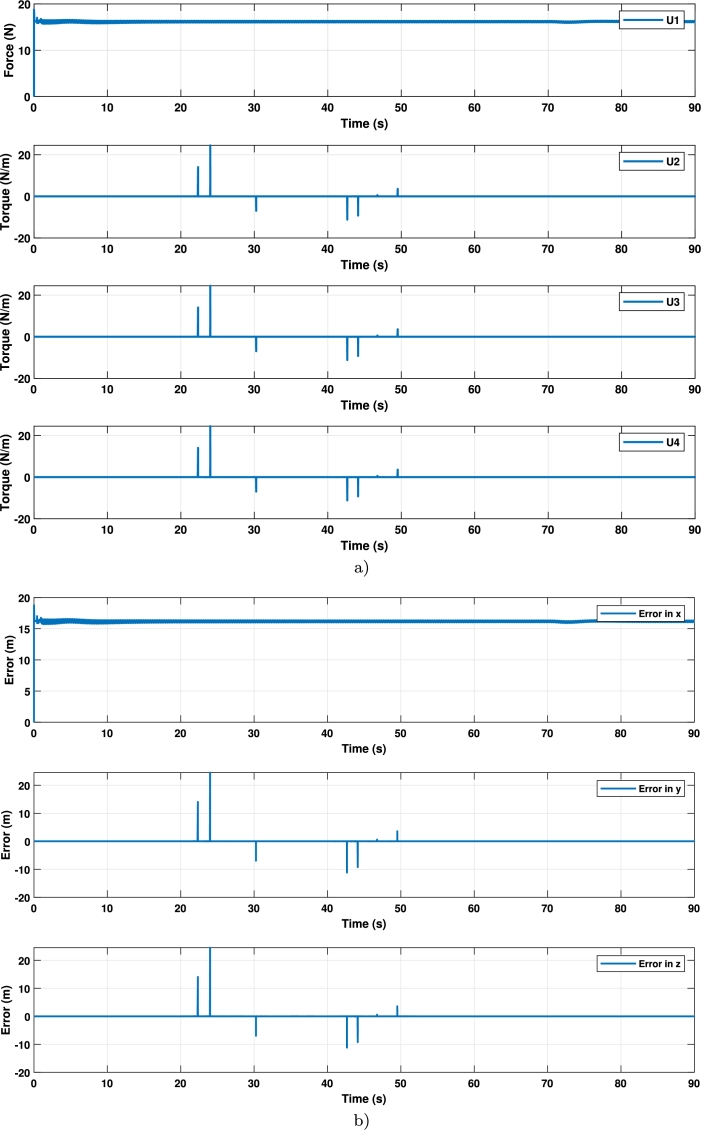
Signals control and errors in the trajectory 1 (**a**) Signals control; (**b**) errors in tracking.

The tracking errors are shown in Fig. 8a) and control signals results of path one are shown in Fig. 8b).

Despite being a simple tracking trajectory, a clear improvement is observed in Fig. 6 when using the adjusted controller. For path 2, a helical type path was chosen, described by Equation (6).

**Figure 9:**
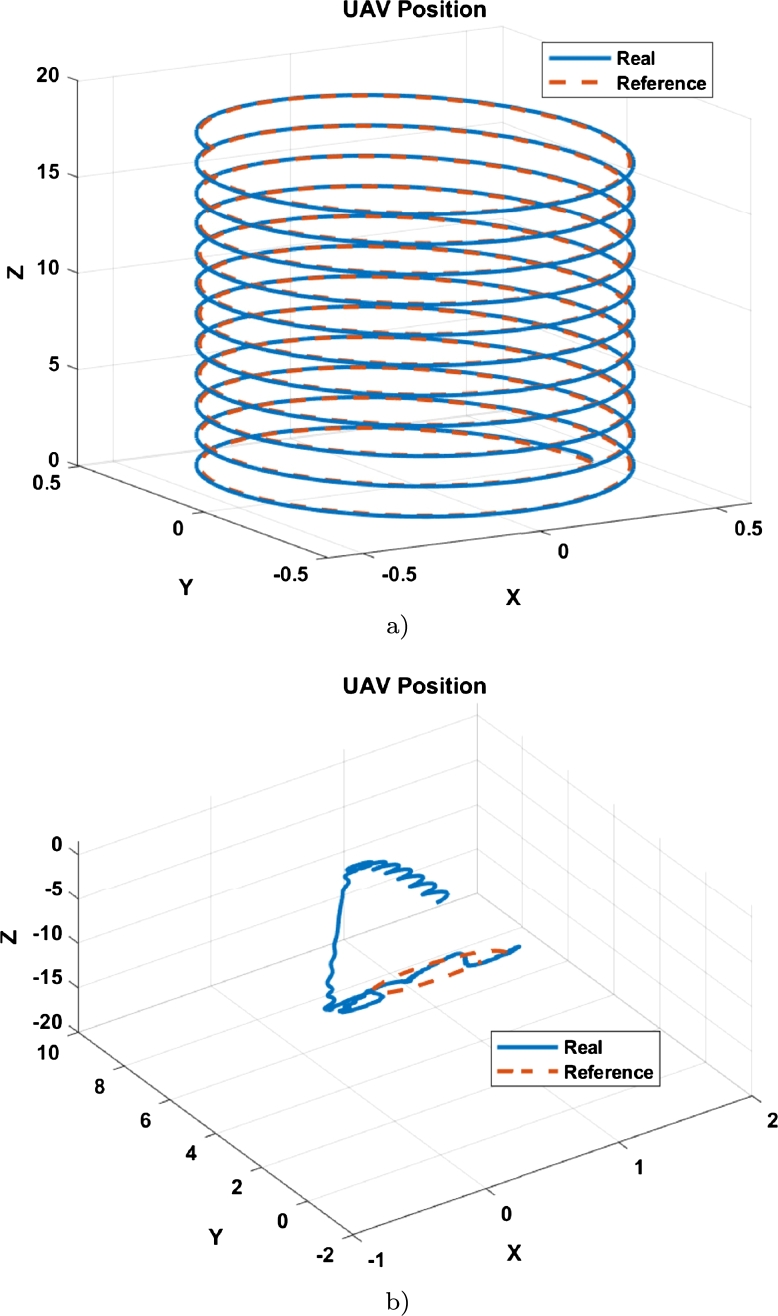
Position of the UAV in tracking the trajectory 2 (**a**) Tracking with tuned controller; (**b**) Tracking with untuned controller.

This trajectory has more difficulty than the first since it has constant motion changes in the x-y plane. The result of the second trajectory is exhibited in Fig. 9 with the tuned controller is show in Fig. 9a) and the untuned controller is depicted in Fig. 9b).

**Figure 10:**
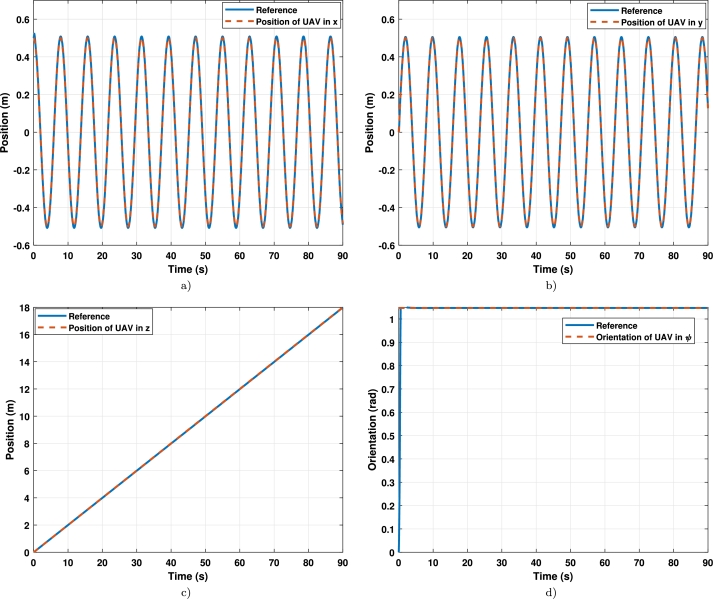
Position per axis of the UAV in tracking the trajectory 2 (**a**) x-axis vs reference; (**b**) y-axis vs reference; (**c**) z-axis vs reference; (**d**) *ψ*-axis vs reference.

The results show that the untuned controller cannot follow the trajectory, and only the first 7 seconds of the trajectory were simulated. The effects of the second tracking trajectory with the tuned controller are observed in detail in Fig. 10, where Fig. 10a) is the axis x, Fig. 10b) is the axis y, Fig. 10c) is the axis z, and Fig. 10d) is the yaw angle.

**Figure 11:**
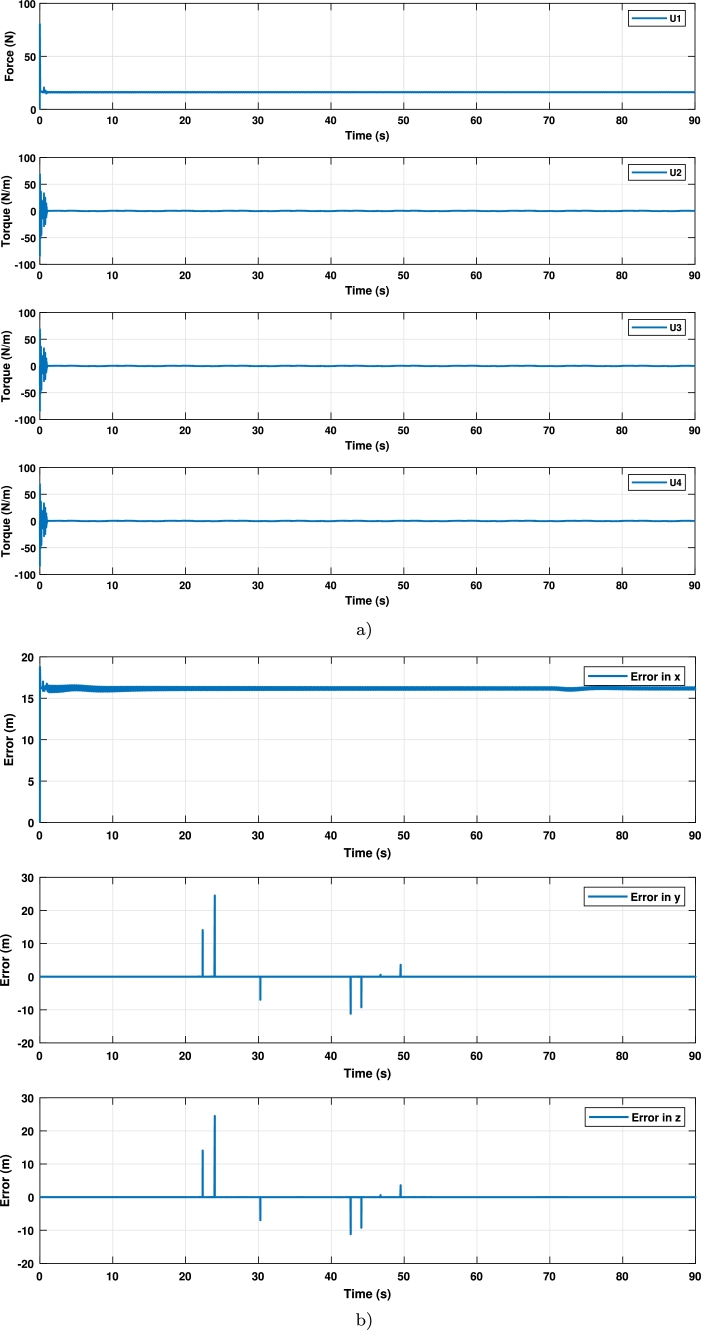
Signals control and errors in the trajectory 2 (**a**) Signals control; (**b**) errors in tracking.

Finally, the torques are displayed in Fig. 11a), and signals control are displayed in Fig. 11b).

It can be seen that the tuned controller has better performance than the untuned controller, as displayed in Fig. 9. This is because the untuned controller could not track the trajectory, while the tuned one could achieve it smoothly.

**Figure 12:**
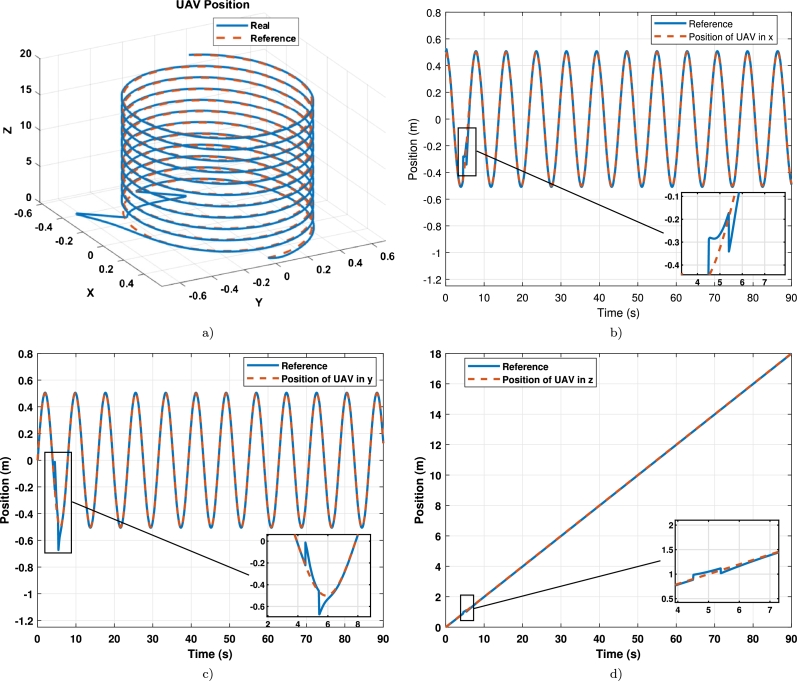
Position per axis of the UAV in tracking the trajectory 2 under disturbance (**a**) x-axis vs reference; (**b**) y-axis vs reference; (**c**) z-axis vs reference; (**d**) *ψ*-axis vs reference.

Disturbances on a UAV are generally gusts of wind. Unlike manipulators, UAVs do not face load changes because they do not interact with the environment. Therefore, a wind gust with random magnitudes on all three axes was added from 4.5 seconds to 5.5 seconds in simulation to trajectory 2 to observe controller performance under typical, expected disturbances. The result of tracking the trajectory of the tuned controller under disturbances is shown in Fig. 12, where Fig. 12a) shows the UAV movement in space, Fig. 12b) displays the trajectory in the x-axis, Fig. 12c) displays the trajectory in the y-axis and Fig. 12d) displays the trajectory in the z-axis.

The Genetic Algorithm found the gains that meet the design requirements of the controller. The design requirements were two characteristics of the dynamic response. The weak points of this method lie in the fact that a globally optimal solution cannot be guaranteed since no metaheuristic algorithm complies with this characteristic. On the other hand, for the correct tuning, it is necessary to know the dynamic model of the plant and the parameters of this model. Depending on the plant it wants to control, this can be complex. Additionally, the proposed method does not contain any procedure to detect combinations or design requirements that may make the system unstable or not physically possible. Therefore, the design parameters must be chosen, considering the physical limits of the plant. Finally, Table 4 was made to expose the differences between our work and other similar investigations. One of the principal differences is the use of dynamic response to calculate the value of the gains.

**Table 4:** Comparison of the proposed tuning system with similar works.

	Variables and methods used					
Work	ts	Mp	Plant	Using of fuzzy logic	Tuning method	Additional variables
Our Work	x	x	UAV	Controller	Genetic algorithm	–
[8]	–	–	DC motor	Gain tuner	Fuzzy	–
[9]	–	–	Active Suspension	Gain tuner	Fuzzy	Unknown nonlinear dynamics
[10]	–	–	UAV	Gain tuner	Fuzzy	Output controller
[11]	–	–	UAV	Controller	Double exponential smoothing	Environment video
[17]	–	–	UAV	Controller	Bad algorithm	RMSE

## 6 Conclusions

In this work, a gains tuning fuzzy controller system was developed, and the gains were added to the fuzzy controller inputs along with a non-linearity of the saturation type to keep the inputs in the operating range. The gains were adjusted with the Genetic Algorithm. The results indicate improved path tracking as path two was completed if the tuned controller was used, and the task was incomplete if the untuned controller was used.

Although other parameters of the fuzzy controller can be adjusted, a greater knowledge of the inputs and outputs of the system is required. When the operating ranges are not precisely known, the method presented in this article can be used to optimize a controller. However, its use depends on knowing the system model and its precision. Finally, the computational cost depends on the parameters of the algorithm and the complexity of the differential equations that describe the dynamic model. Therefore, in some cases, tuning by this method is not feasible.

## CRediT authorship contribution statement

**Omar Rodríguez-Abreo:** Writing – review & editing, Writing – original draft, Visualization, Validation, Supervision, Software, Resources, Project administration, Methodology, Investigation, Funding acquisition, Formal analysis, Data curation, Conceptualization. **Juvenal Rodríguez-Reséndiz:** Writing – review & editing, Writing – original draft, Methodology, Investigation. **A. García-Cerezo:** Writing – review & editing, Writing – original draft, Supervision, Conceptualization. **José R. García-Martínez:** Writing – review & editing, Writing – original draft, Investigation.

## Declaration of Competing Interest

The authors declare that they have no known competing financial interests or personal relationships that could have appeared to influence the work reported in this paper.

## Data Availability

Data will be made available on request.
